# Application of the Hydrophilic Interaction Liquid Chromatography (HILIC-MS) Novel Protocol to Study the Metabolic Heterogeneity of Glioblastoma Cells

**DOI:** 10.3390/metabo14060297

**Published:** 2024-05-23

**Authors:** Jakub Šofranko, Eduard Gondáš, Radovan Murín

**Affiliations:** 1 Department of Medical Biochemistry, Jessenius Faculty of Medicine in Martin, Comenius University in Bratislava, Mala Hora 4D, 036 01 Martin, Slovakia; 2 Department of Pharmacology, Jessenius Faculty of Medicine in Martin, Comenius University in Bratislava, Mala Hora 4D, 036 01 Martin, Slovakia

**Keywords:** glioblastoma, HILIC, LC-MS, metabolomics, amino acid, metabolic heterogeneity

## Abstract

Glioblastoma is a highly malignant brain tumor consisting of a heterogeneous cellular population. The transformed metabolism of glioblastoma cells supports their growth and division on the background of their milieu. One might hypothesize that the transformed metabolism of a primary glioblastoma could be well adapted to limitations in the variety and number of substrates imported into the brain parenchyma and present it their microenvironment. Additionally, the phenotypic heterogeneity of cancer cells could promote the variations among their metabolic capabilities regarding the utilization of available substrates and release of metabolic intermediates. With the aim to identify the putative metabolic footprint of different types of glioblastoma cells, we exploited the possibility for separation of polar and ionic molecules present in culture media or cell lysates by hydrophilic interaction liquid chromatography (HILIC). The mass spectrometry (MS) was then used to identify and quantify the eluted compounds. The introduced method allows the detection and quantification of more than 150 polar and ionic metabolites in a single run, which may be present either in culture media or cell lysates and provide data for polaromic studies within metabolomics. The method was applied to analyze the culture media and cell lysates derived from two types of glioblastoma cells, T98G and U118. The analysis revealed that even both types of glioblastoma cells share several common metabolic aspects, and they also exhibit differences in their metabolic capability. This finding agrees with the hypothesis about metabolic heterogeneity of glioblastoma cells. Furthermore, the combination of both analytical methods, HILIC-MS, provides a valuable tool for metabolomic studies based on the simultaneous identification and quantification of a wide range of polar and ionic metabolites—polaromics.

## 1. Introduction

Glioblastoma is a highly malignant brain tumor that is characterized by its invasive growth and resistance to current therapies [1,2]. Metabolomics, the study of small molecule metabolites in biological systems, has emerged as a powerful tool for understanding the metabolic alterations in glioblastoma cells and identifying potential therapeutic targets. Studies in metabolomics, including those focusing on glioblastoma, have identified several altered or dysregulated metabolic pathways in comparison to healthy cells [3,4,5,6,7,8]. Examples of these alterations include changes in glucose and amino acid metabolism. Glioblastoma cells have been shown to rely on glucose and several amino acids to support their high energy demands [9,10,11,12,13,14], as well as to utilize their carbon skeletons for anabolic processes such as lipid [15,16] and nucleotide synthesis [17]. Despite the genetic and cellular heterogeneity observed in glioblastoma cells within tumors [4], as well as heterogeneity in their metabolism [18,19,20], targeting metabolic pathways has emerged as a promising therapeutic strategy. Therefore, further studies focusing on and revealing the metabolic aspects of cancer cells could provide valuable knowledge. 

Recent methodological approaches capitalize on advanced analytical techniques and methods capable of sensitive, comprehensive, and robust analysis. In this regard, liquid chromatography (LC) is a state-of-the-art analytical technique used for the separation, purification, or concentration of compounds from complex mixtures [21]. Chromatographic retention of molecules is based on differences in their physical and chemical properties, such as polarity and solubility. The majority of LC applications currently exploit reliable and reproducible separation of non-polar compounds through reversed-phase LC [22]. However, since a significant part of metabolites bear polar or ionic group, their separation by reversed-phase liquid chromatography often requires chemical modification through derivatization or relies on ion pairing [23,24]. Alternative to separation of polar compounds could be ion-exchange chromatography, but this technique requires high concentrations of buffers in the mobile phase, which could have negative effects on the ionization source [25]. Supercritical fluid chromatography, which uses supercritical CO_2_ as the mobile phase, is becoming popular for metabolomics analysis in recent years. Unfortunately, there are instrumental limitations for this method, which means it cannot be applied in every laboratory equipped with a mass spectrometer [26]. Therefore, it seems to be demanding to introduce more simple methods for the separation of polar and ionic compounds by LC without prior derivatization, if possible [27]. 

Indeed, recent focus on the practical application of polar and ionic resins as the stationary phase has allowed for more effective separation of polar and ionic metabolites [28]. In this respect, hydrophilic interaction chromatography (HILIC) has become more widely used in metabolic studies, due to its capability to separate polar and ionic compounds such as amino acids, sugars, nucleotides, and peptides. Combining HILIC with the mass spectrometry (MS) could provide the method with high sensitivity and specificity. HILIC-MS can be exploited for quantitative analysis of a large number of polar and ionic metabolites simultaneously, and even a small volume of biological sample is needed [29]. Indeed, HILIC-MS has also enabled the development of high-throughput and automated analytical methods for the analysis of complex samples and can provide “polaromics” data in field of metabolomics (Šofranko et al. in preparation). 

Therefore, we adopted HILIC-MS for simultaneous quantitative analysis of polar and ionic compounds present in biological matrixes such as culture media or cell lysates. We utilized this method in a metabolic study focused on identification putative metabolic differences between two types of cultured glioblastoma cell lines. The adopted HILIC-MS method allows us to quantify about 150 metabolites in a single run without derivatization. By applying this method to analyze the culture media and cell lysates, we revealed differences between two types of glioblastoma cells in metabolizing some of the substrates present in their media, as well as differences in the levels of their intracellular metabolites. These results confirm the presence of metabolic heterogeneity among glioblastomas and demonstrate the potential of employing HILIC-MS analysis for metabolic profiling of cultured cells and for identifying potential targets for either new therapeutic or diagnostic strategies. 

## 2. Materials and Methods

Acetonitrile, LC-MS grade with purity ≥ 99.9%, was obtained from Honeywell (Charlotte, NC, USA). Dulbecco’s modified Eagle’s medium (DMEM), Dulbecco’s phosphate buffered saline (DPBS) without Ca^2+^ and Mg^2+^ and fetal bovine serum were purchased from Thermo Fisher Scientific (Waltham, MA, USA). Antibiotics, penicillin G, and streptomycin sulfate were from PAA Laboratories GmbH (Cölbe, Germany). The DC protein assay kit was purchased from Bio-Rad (Hercules, CA, USA). Ammonium formate, for LC-MS, was purchased from Honeywell (Charlotte, NC, USA) and formic acid from Santa Cruz Biotechnology, (Dallas, TX, USA). All other chemicals, including analyte standards as well as isotopically labeled standards, and materials were sourced from Sigma-Aldrich (St. Louis, MA, USA) in highest available purity. Ultra-pure water generated by Milli-Q instrument (Merck, Boston, MA, USA), was used for all experiments. The resistivity of water was at least 18.2 MΩ·cm at 25 °C and total organic carbon content ≤ 5 ppb.

### 2.1. Preparation of External Standards Spiked with Internal Standard

External standards were dissolved in water at a mass concentration of 1 g/L. A maximum of 10 individual standard solutions were pooled together to create the mixtures with a mass concentration of each molecule in the mixture at a level of 100 mg/L. These mixtures of ten compounds were again pooled together and further diluted to the desired mass concentrations. The final dilution of mixture of all external standards was performed in ratio 1:9 with acetonitrile, which was pre-spiked with internal standard, i.e., isotopically labeled leucine (^13^C_6_, ^15^N). 

### 2.2. Collection of Cell Culture Media and Lysates

Two types of glioblastoma cell lines, T98G (ATCC-CRL-1690, Manassas, VA, USA) and U-118 (ATCC-HTB-15, Manassas, VA, USA) MG, were cultured in Dulbecco’s modified Eagle’s medium (DMEM) supplemented with 10% (*v*/*v*) fetal bovine serum, 100 I.U./mL penicillin, and 0.1 mg/mL streptomycin sulfate under conditions as previously described [10,30,31]. The cell culture medium was refreshed 24 h prior to the experiment. As a control, an aliquot of fresh medium was stored for further LC-MS analysis to establish the initial level of metabolites at time 0. The media and lysates were either further analyzed or stored for short time at −20 °C.

### 2.3. Media and Lysates Preparation for LC-MS Analysis

For medium analysis, 10 µL of culture medium was added to 200 µL of ice-cold mixture of acetonitrile enriched with 1 mg/L of isotopically labeled leucine (^13^C_6_, ^15^N). After brief vortexing, the mixture was kept at −20 °C for 30 min. Subsequently, samples were clarified by centrifugation at 10,000× *g* for 10 min. Supernatants were transferred into new tubes and either directly used for LC-MS analysis or stored at −20 °C for short time prior further analysis.

To obtain samples from cellular lysates, adhered cells were rinsed twice with PBS and subsequently lysed in 200 µL of ice-cold lysis solution composing from 90% (*v*/*v*) acetonitrile and 10% (*v*/*v*) H_2_O, spiked with 1 mg/L of isotopically labeled leucine (^13^C_6_, ^15^N). After scraping the cells with a rubber scrapper, the suspension was quantitatively transferred into tubes. The supernatants prepared by centrifugation of lysates at 10,000× *g* for 10 min were collected and further analyzed by LC-MS or stored for a short time at −20 °C. The pellets were air-dried and subsequently dissolved for protein estimation. 

### 2.4. LC-MS Analysis

Four chromatographic columns were tested for the chromatographic separation of polar and ionic compounds: Sequant^®^ ZIC^®^-cHILIC (3 µm, 100 A, 150 × 2.1 mm; Sigma Aldrich, Steinheim, Germany), Sequant^®^ ZIC^®^ pHILIC (5 µm, 150 × 2.1 mm; Sigma Aldrich, Germany), YMC-Triart-Diol-HILIC (1.9 µm, 150 × 2.1 mm; YMC, Dinslaken, Germany), and Raptor Polar X (2.7 µm, 100 × 2.1 mm; Restek, Bellefonte, PA, USA). During chromatographical separation the columns were tempered to 40 °C except for Raptor Polar X, for which 30 °C was applied. 

For separation on Sequant^®^ ZIC^®^ cHILIC and pHILIC columns, one of the mobile phases consisted of 20 mM ammonium formate solution in water (mobile phase A) and the second consisted of 100% acetonitrile (mobile phase B; Honeywell, USA). Initial conditions were 90% (*v*/*v*) of acetonitrile to 10% (*v*/*v*) of ammonium formate in water at a flow rate of 0.2 mL/min, and the sample injection volume was 2 µL. The gradient then changed to 20% (*v*/*v*) acetonitrile over 24 min, followed by a return to initial conditions over 6 min, with an additional 2 min for column conditioning. 

Chromatographic separation performed on YMC-Triart-Diol-HILIC used the same mobile phases as for separation on ZIC^®^ columns. Gradient started with the same initial conditions, decreased to 40% mobile phase B (*v*/*v*) over 13 min and then returned to original conditions in 7 min. Columns were equilibrated with 90% B for an additional 3 min. Total flow was 0.2 mL/min. The sample injection volume was 2 µL.

The composition of mobile phases for separation on the Raptor Polar X column differed from the previous ones. Mobile phase A consisted of 0.5% formic acid in water, while mobile phase B consisted of acetonitrile and 20 mM ammonium formate in water, with a pH of 3, in a ratio of 9:1. The separation was executed using the following gradient: initiation with 88% of phase B maintained for 3 min, followed by a transition to 30% B over 4.5 min, then an abrupt return to 88% of B. The column was then equilibrated for an additional 2 min under initial conditions. The total flow rate was 0.5 mL/min. The sample injection volume was 2 µL.

LC-MS analysis was performed on LC-MS setup (Shimadzu, Kyoto, Japan) with the specification already described [32]. The settings were as follows: the ion spray voltage was set to 4.5 kV for positive and −3.5 kV for negative mode. Heat block and capillary desolvation line were both at 200 °C. Nitrogen nebulizing gas flow was at 1.5 L/minute and drying gas pressure was set at 200 kPa. The ion accumulation time in the ion trap was set to 40 ms, and the range for data acquisition was 100–600 *m*/*z*. Data were acquired in both positive and negative mode simultaneously using LabSolutions v 3.81 software (Shimadzu).

### 2.5. Processing of Spectra 

Acquired spectra were directly converted to mzXML format using LabSolutions v 3.81 post-run analysis and imported to Skyline 23.1. (MacCross Lab Software), an open source software for LC-MS data processing (https://skyline.ms (accessed on 1 December 2023)) [33]. Peaks of individual metabolites were extracted from MS scans to obtain information such as mass-to-charge ratio (*m*/*z*), retention time (t_R_), areas under the peak (AUP), and the full width at half maximum (FWHM) of the peak. Metabolite identification in samples was based on confirmation of its retention time, with maximum deviation of ±0.1 min, and *m*/*z* by compounds from our internal library, acquired using analytical standards, or with online libraries such as Pubchem (https://pubchem.ncbi.nlm.nih.gov/ (accessed on 1 February 2024)) and mzCloud (https://www.mzcloud.org/ (accessed on 1 February 2024)). Analytes were not further fragmented because we considered identification according to retention time and *m*/*z* as sufficient, as almost every identified metabolite is included in our internal database. Peak resolution was calculated using retention time of analytes and the full width at half maximum of their peaks, according to the following formula:

Rs = 1.18 × [(t_R2_ − t_R1_)/(w_0.5h1_ + w_0.5h2_)]

where t_R2_ is retention time of later eluting analyte, and t_R1_ is retention time of early eluting analyte. The parameter w_0.5h1_ represents a width at half maximum of peak of early eluting analyte and w_0.5h2_ is a width at half maximum of peak of later eluting analyte [22].

### 2.6. Quantification of Metabolites

For quantification purposes, the AUP of each metabolite was normalized by dividing this AUP with the AUP of internal standard—^13^C_6_ ^15^N leucine. Subsequently, the concentration of the analyte was calculated using external standards, with a known concentration, for which areas were normalized as well, where external standards are all compounds included in Table 1, except for fatty acids—cervonic acid, γ-linolenic acid, linoleic acid, palmitic acid, and stearic acid and carnitines except for octanoylcarnitine [34]. The external standards were the same compounds as the metabolites, dissolved in acetonitrile and water (9:1 ratio) and analyzed in a different injection. To obtain the same mass concentration of internal standard (1 μg/mL) in the samples and external standards, the internal standard was dissolved using the whole volume of acetonitrile. For injection reproducibility control, precision of internal standard of each injection was calculated, which is expressed as relative standard deviation (RSD) of its AUP. 

### 2.7. Protein Quantification

Protein concentration in individual samples was determined using a commercially available DC protein assay kit, which is based on a modified Lowry assay [35]. The bovine serum albumin was used as a standard protein for generation of a calibration curve.

### 2.8. Statistical Analysis

Data are reported as mean ± standard error of the mean (SEM) from three independent trials. Statistical analysis was performed using the web browser application Metabolite AutoPlotter 2.6. (https://mpietzke.shinyapps.io/AutoPlotter/ (accessed on 1 February 2024)) [36]. Comparison between two groups, different cell lines, was performed using Student’s *t*-test, and a *p*-value lower than 0.05 was considered statistically significant, where one asterisk means *p* < 0.05, two asterisks *p* < 0.01, three asterisks *p* < 0.001 and four asterisks *p* < 0.0001. To construct PCA plots, amounts of substances per milligram of proteins, for all identified and quantified compounds in samples were used. Heatmap of metabolites in culture medium was constructed from fold change of the amount of substance in a medium without cells and medium after incubation. For stearic and palmitic acid, the fold change was calculated from ration of their AUP to AUP of IS. Secondly, a heatmap depicting metabolites in lysates represents the estimated average amount of substance in micromole per mg of lysate proteins. Heatmaps and PCA were created using web browser application MetaboAnalyst 6.0 (https://dev.metaboanalyst.ca/MetaboAnalyst/ (accessed on 1 February 2024)) [37]. 

To monitor any analytical discrepancies that arise during sample preparation and data collection phases, we used medium without cells—DMEM enriched with 10% (*v*/*v*) fetal bovine serum. Three independent samples of this medium were prepared, as described in Media for analysis and lysates preparation, and injected to LC-MS. For each identified analyte we estimated the precision, which is expressed as RSD of the measurement of three independent samples (Table S1, Figure S1). In agreement with the previously published limit for exclusion of metabolites, the limit was set to precision lower than 30% for the metabolite not to be excluded [38,39,40]. In addition, we used the software Metabolite AutoPlotter feature “Quality control” to investigate putative outliers (Figure S1), for which the fold-change is below 0.5 or above 2. These metabolites should be excluded from analysis [36]. If there is a metabolite that does not meet both of these requirements, we would exclude it from the study.

## 3. Design and Testing of LC-MS

In our effort to separate and quantify the polar and ionic molecules related to cellular metabolism, we applied mass spectrometry detection techniques to compounds eluted from chromatographic columns with zwitterionic stationary phases. Four columns: Raptor Polar X, ZIC^®^ pHILIC, ZIC^®^ cHILIC, and Diol-HILIC underwent examination to discern their efficacy in separating selected compounds. Process of HILIC column selection was simplified according to our research demands. After column selection, we tested method reliability and applied it to biological samples.

**Figure 1:**
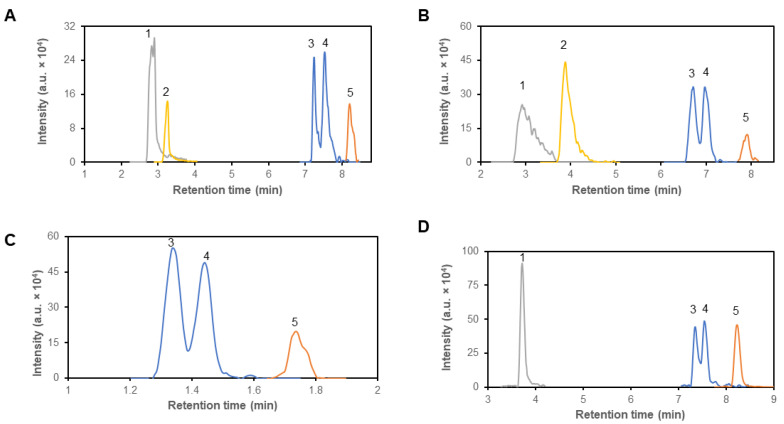
Representative chromatogram of of α-ketoisocaproic acid (1), α-ketoisovaleric acid (2), isoleucine (3), leucine (4) and valine (5), on four different columns—Sequant^®^ ZIC^®^ cHILIC (**A**), Sequant^®^ ZIC^®^ pHILIC (**B**), Raptor polar X (**C**) and YMC-triart Diol-HILIC (**D**). Compounds were detected according to their *m*/*z* values (*m*/*z* = 129.0582, grey line; *m*/*z* = 115.0428, yellow line; *m*/*z* = 132.1019, blue line; *m*/*z* = 118.0863, orange line) and retention times. Mass concentration of all compounds was 1 μg/mL.

A primary criterion for column selection was its capability to effectively separate structural isomers of branched-chain amino acids, i.e., leucine and isoleucine (Figure 1), along with pairs of amino acids sharing similar mass, like glutamine and lysine (Table 1). Since the *m*/*z* difference between glutamine and lysine could be sufficient to be separated in a high-resolution mass spectrometer, we would like to highlight the need of their separation in the case of using a low-resolution mass spectrometer. There it could pose a problem with its identification and subsequent quantification, if not separated properly. Notably, all four columns provided capability to separate lysine and glutamine isomers, and in addition, they separated molecules of leucine and isoleucine to a certain extent (Figure 1). Based on the obtained values of parameters for the peak of leucine and isoleucine, the peak resolution values could be calculated (Table 2). The highest peak resolution value was established for separation on the Sequant^®^ ZIC^®^-cHILIC column and a value of 1.28 reached.

In subsequent testing, we assessed the columns abilities to distinguish between branched-chain 2-oxo acids: α-ketoisocaproic acid (KIC), α-keto-β-methylvaleric acid (KMV) and α-ketoisovaleric (KIV), cognate 2-oxo acids of leucine, isoleucine, and valine, respectively. Only ZIC^®^ columns were able to retain all branched-chain 2-oxo acids, but none of the tested columns separated molecules of KIC and KMV. YMC-Triart column was able to retain only KIC and KIV (Figure 1). 

**Figure 2:**
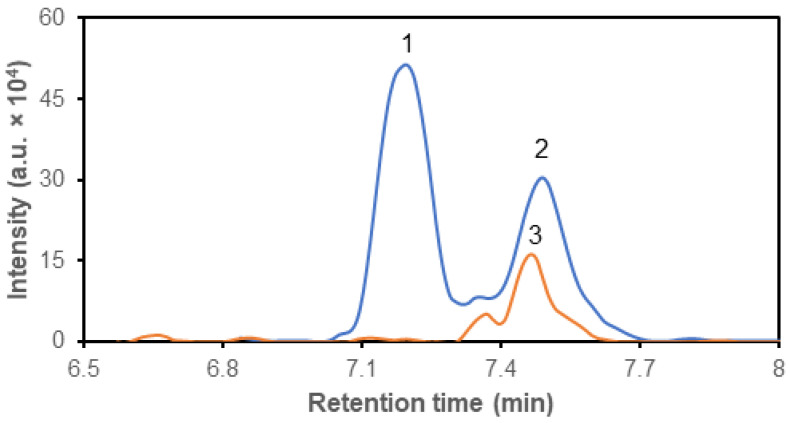
Representative chromatogram of isoleucine (1, blue), leucine (2, blue) and isotopically labeled ^13^C_6_,^15^N-leucine (3) separation on Sequant^®^ ZIC^®^ cHILIC column. The *m*/*z* values (*m*/*z* = 132.1019, blue line; *m*/*z* = 139.1191, orange line) were used for identification after elution.

Based on these inclusion criteria, the Sequant^®^ ZIC^®^ cHILIC column was selected for further experiments. Standard solutions and their mixtures, prepared from purchased compounds (Table 1), were supplemented with ^13^C_6_,^15^N-leucine as an internal standard (Figure 2). Subsequently, the acquired spectra were processed using Shimadzu software or Skyline to derive values for the area under the curve (AUC). Calibration curves were generated for selected metabolites, and estimated values of linearity (R^2^), linearity range and precision were determined (Table 3).

The majority of tested polar and ionic compounds were successfully separated on the cHILIC column and subsequently detected by MS. Retention times and *m*/*z* values were assigned to each detected compound, laying the groundwork for metabolic analysis.

### 3.1. Identification and Quantification of Metabolites in Media

**Figure 3:**
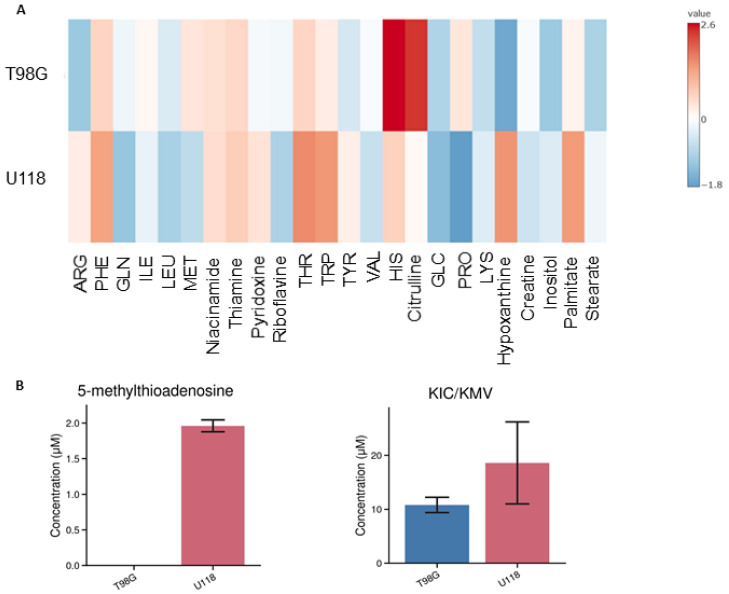
Metabolic changes in culture medium of two glioblastoma cell lines (T98G and U118) incubated for 24 h. (**A**) Heat map, where data are expressed as average of the fold change of metabolites in medium without cells and metabolites in medium after incubation. Metabolites depicted with a blue color are taken up from the medium and metabolites depicted with a red color are released into the medium. (**B**) Increase in 5-methylthioadenosine and 4-methyl-2-oxopentanoate/3-methyl-2-oxopentanoate in culture medium after incubation. These are depicted here in separate graphs because it was not possible to calculate the fold change, since the two were not detected in the medium without cells. Blue color represents the T98G cell line, and red represents the U118 cell line. Numbers of replicates for each sample is three (*n* = 3). Error bars are expressed as SEM.

The method was applied on culture media from two lines of glioblastoma cell lines, i.e., T98G and U118. The change of the media composition was analyzed after 24-h cultivation. By LC-MS analysis, a total of 26 metabolites were quantified in culture media (Figure 3A).

Out of the detected metabolites, there were ten metabolites whose level in the media varied dependently on the type of the glioblastoma cell. The cells affected the levels of these metabolites due to their uptake or release. The specific uptake and release have been calculated by dividing the amount of substance in medium by the number of proteins in cell lysates and time of incubation.

**Figure 4:**
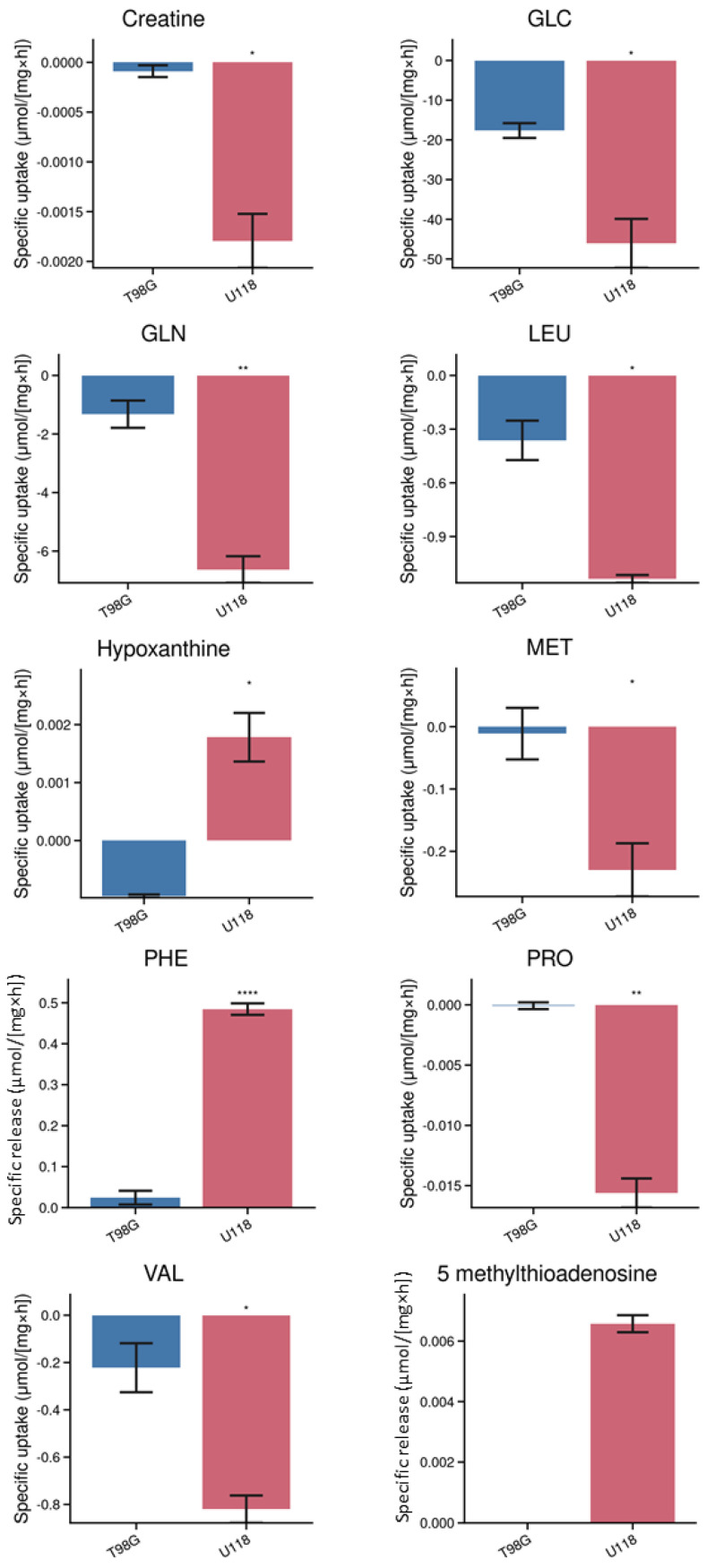
Changes of metabolites in culture medium of T98G (blue) and U118 (red) cell lines. Bar graphs of metabolites indicating specific uptake or release after 24 h of incubation. Numbers of replicates for each sample is three (*n* = 3). Error bars are expressed as SEM. One asterisk means *p* < 0.05, two asterisks *p* < 0.01 and four asterisks *p* < 0.0001.

**Figure 5:**
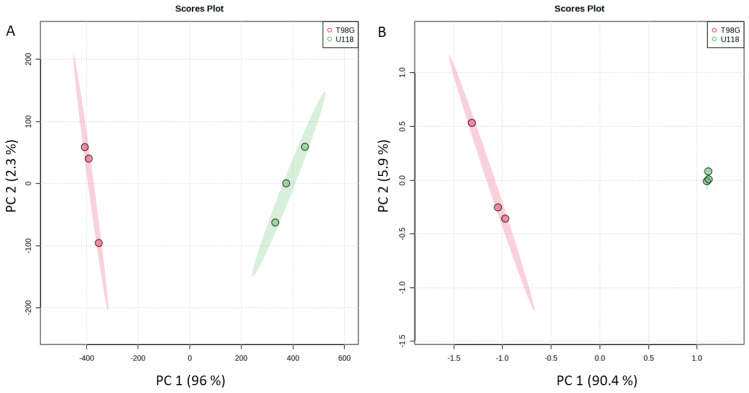
Principal component analysis (PCA) score plot of three replicates of two different groups, from all metabolites detected in collected culture media (**A**) or cell lysates (**B**) of both types of glioblastoma cells, T98G (red) or U118 (green).

The differences between the tested types of glioblastoma cells are capable of removing or releasing creatine, glucose, leucine, glutamine, proline, methionine, valine, 5-methylthioadenosine, phenylalanine and hypoxanthine. We identified ten compounds, whose levels in media differed significantly after incubation depending on the type of glioblastoma cells (Figure 4). For example, U118 glioblastoma cells tend to excrete 5-methylthioadenosine in contrast to T98G cells, which released a substantial amount of citrulline. The estimated differences for metabolic capacity of U118 and T98G cells were tested by PCA analysis, whose results clearly indicate distinctions between their metabolisms (Figure 5A).

### 3.2. Identification and Quantification of Metabolites in Lysates

**Figure 6:**
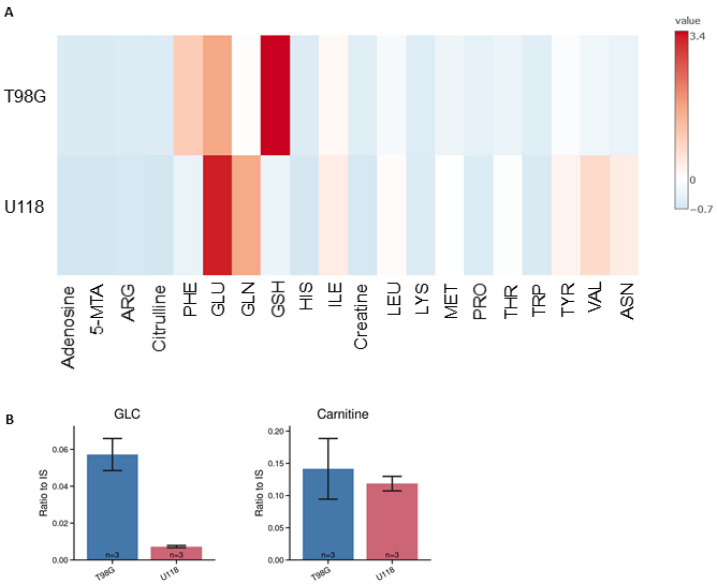
Intracellular content of metabolites in two types of glioblastoma cells, T98G and U118. (**A**) Heatmap represents the estimated average amount of substance in micromole per mg of lysate proteins. (**B**) The relative levels of glucose and carnitine are presented on separate graphs since their content, in lysates, was quantified only relatively as the signal of the compound to the peak area of the internal standard. Two cell lines were compared: T98G (blue) and U118 (red). Relative quantification is expressed as a ratio of the peak area of analyte to the peak area of the internal standard. Numbers of replicates for each sample is three (*n* = 3). Error bars are expressed as SEM.

We also applied the current method for quantification of intracellular metabolites. The LC-MS analysis of cell lysates allowed for quantification of 20 metabolites (Figure 6), whose levels were standardized to protein content of cells lysates.

**Figure 7:**
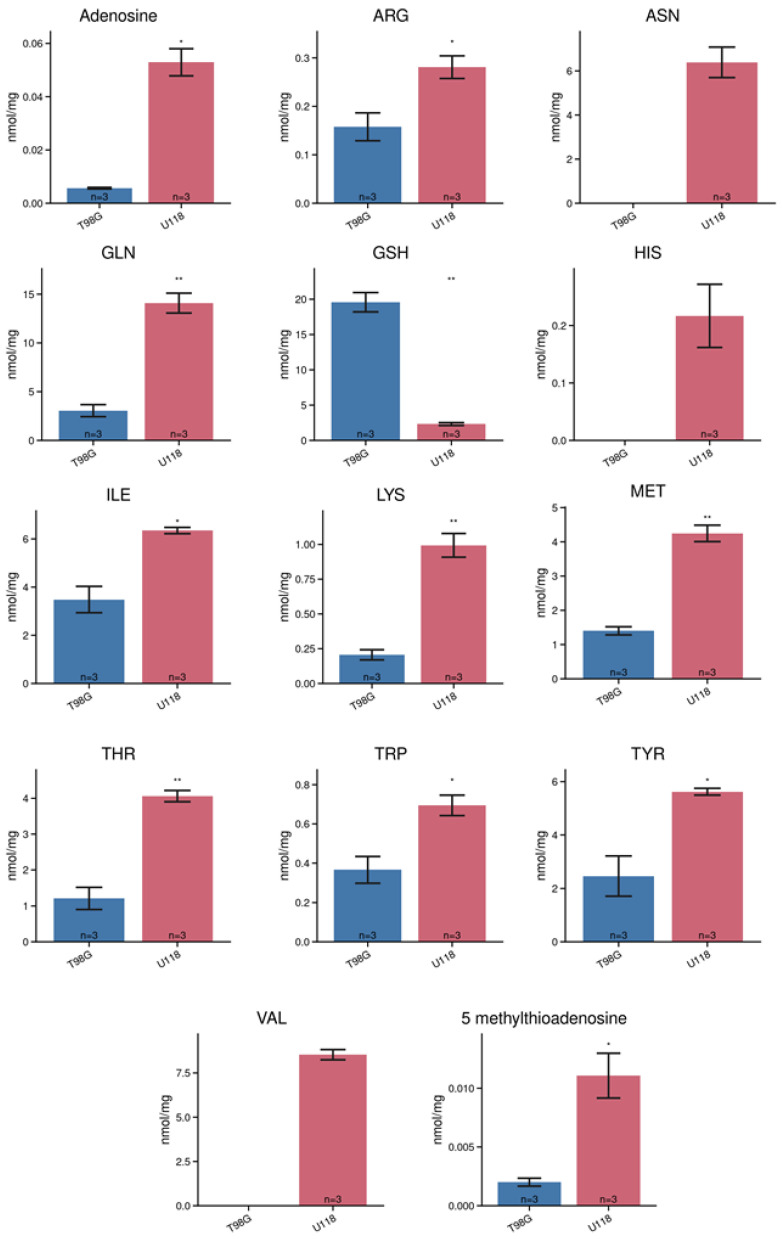
Estimation of the intracellular levels of metabolites in cultured T98G (blue column) and U118 (red) glioblastoma cells by LC-MS. The metabolites were quantified in lysates derived from culture cells. Bar graphs representing the molar amount of substance standardized per one mg of lysates proteins. Numbers of replicates for each sample is three (*n* = 3). Error bars are expressed as SEM. One asterisk means *p* < 0.05, two asterisks *p* < 0.01.

Out of them, the intracellular content of 14 metabolites differed between the tested types of glioblastoma cells (Figure 7). Asparagine, histidine, and valine were not detected in lysates of the T98G cell line. Such differences were used to generate the PCA score plot (Figure 5B) that clearly illustrates the metabolomic differences between U118 and T98G glioblastoma cells.

## 4. Discussion

In the present study we exploited the possibility of the newly adapted LC-MS method for quantification of ionic and polar compounds in culture media and cell lysates of cultured human glioblastoma cells—polaromics.

An advantage of HILIC-MS is the ability to retain a wide range of polar and ionic compounds without prior derivatization and their separation from nonpolar metabolites. Concentration of salts in the mobile phase does not need to be high in order to effectively elute analytes hence it does not affect the sensitivity or parts of ionization source negatively. Another benefit is the simple sample preparation which does not need any complicated extraction technique that can bring additional errors to the process and could also be time consuming. There is also no need to add ion-pairing reagents to the mobile phase, such as higher alkyl amines, which are often used for separation of polar metabolites on RP columns. This can lead to suppression of one of the polarity modes and thus it is unable to analyze the sample in both polarity modes at the same time and it could lead to contamination or damage to MS parts [27]. With all advantages comes of course also some disadvantages. Elution of less polar compounds, other than lipids, could be early and thus separation from the matrix may not be effective. Furthermore, peaks eluting from HILIC could be often broad or asymmetric. During the interaction of analytes with the stationary phase, secondary electrostatic interactions may occur, hence the separation mechanism is not fully understood and this may have an impact on method optimization [41].

Mass spectrometry is capable of identifying molecules, according to their mass to charge ratio, and subsequent quantification, even of low concentrations of metabolites in samples [42]. In this respect, there are many MS techniques that are frequently used for metabolomic studies. The most common ionization sources used are electrospray ionization (ESI), atmospheric pressure chemical ionization or atmospheric pressure photoionization. Each of the named sources preferably ionize different types of molecules. ESI is the most used and ionizes mostly hydrophilic to medium polar molecules but is less effective on non-polar compounds. The latter two ionize mostly non-polar molecules and thus make good complementary technique to ESI [43,44]. Another feature that can be different in MS-based methods are mass analyzers. Currently, analyzers are mostly used in tandem, with two or more analyzers combined. Most commonly, either triple-quadrupoles (QQQ) or analyzers that provide high resolution such as quadrupole time-of-flight (Q-TOF) or ion trap time-of-flight (IT-TOF) are used. Triple-quadrupoles are more sensitive, but data needs to be collected from narrow user-specified *m*/*z* windows. On the other hand, high resolution mass spectrometers, as the one used by us, operating in full scan modes, can provide wider coverage of metabolites or can help identify molecules with adducts or multiple-charged ions, that would not be expected [45,46].

The results of the LC-MS analysis revealed that glioblastoma cells T98G and U118 differ in their capability to withdraw some of the compounds from culture media and to release metabolites into their milieu.

HILIC-MS is commonly used for the analysis of polar metabolites, such as amino acids, nucleotides, sugars, and organic acids. HILIC-MS has found numerous applications in various fields, including pharmaceuticals, proteomics, metabolomics, and environmental science. In metabolomics studies, it is becoming a tool for the quantification of polar or ionic molecules in biological samples, characterization of metabolic nuances and for the identification of putative biomarkers and drug metabolites. We tested around 150 polar and ionic compounds of interest (Table 2) and evaluated the possibility of their separation on polar columns and subsequent detection and quantification by MS. Based on the previously published results that revealed the capability of glioblastoma cells to readily dispose from culture media glucose and branched-chain amino acids [3,5,9,10,11,12,13,14,17], we focused on the possibility to separate, detect and quantify amino acids in biological matrices by LC-MS. Since leucine and isoleucine are constitutive isomers with identical *m*/*z*, we selected for LC the separation cHILIC column [10], which is capable of sufficiently separating these two amino acids. The cHILIC separation leads to elution of leucine and isoleucine molecules in two discrete peaks for molecules with identical *m*/*z* values but with different retention times (Figure 1A and Figure 2). For the definite estimation of the separation capacity of the column, we used mixtures consisting of isoleucine and leucine molecules, which in their structure contained lighter isotopes ^12^C and ^14^N or heavier ^13^C and ^15^N. The additional advantage of separation on HILIC column is that the compounds are separated on presence of polar and charged moieties of molecules that allows for separation of compounds without prior derivatization [21,22,29].

The analysis of the culture media by the presented LC-MS protocol was sufficiently sensitive to quantify 26 metabolites and to estimate their specific metabolic rates. In addition, the number of compounds was quantified in cell lysates. Our presented results provide evidence that in addition to common metabolic features among tested types of glioblastoma cells, there are metabolites whose quantity is altered depending on the investigated type of glioblastoma cells. We identified 5-methylthioadenosine, proline, methionine and hypoxanthine, as the compounds whose metabolism are most different. Uptake rates of branched chain amino acids—leucine and valine—were significantly higher in the U118 cell line. Valine was also below the limit of quantification inside of T98G cells which can be correlated with its lower uptake rates, and it can be pointed out that U118 cells rely more on the metabolism of this essential amino acid. Same circumstances are here for methionine and glutamine since their uptake rates are higher in the U118 cell line, and their content in lysates is higher as well. Both cell lines have a high demand for glutamine, since it is a vital amino acid for glioma growth because it is source of carbon for the citric cycle or nitrogen for the biosynthesis of nucleosides [47]. The amount of proline has almost not changed in the medium of T98G unlike in the culture medium from the other cell line. It was shown, that proline metabolism is associated with cancer metastasis and during glucose deprivation of astrocytes, it compensates as a substrate for ATP production, which refers to an important anaplerotic role [48,49]. In contrast, 5-MTA was released into culture medium of the U118 cell line but not into the culture medium of the T98G cell line and the intracellular amount was also higher in U118. It is hypothesized that 5-MTA has a positive effect on tumor cell proliferation, which could explain the differences in uptake of some of the metabolites from the medium between these two cell lines. Where uptake of substrates in the U118 cell line was in general higher than in T98G, it could be a sign of higher demand to fulfil the metabolism needs [50].

Only the metabolite whose amount is higher in the T98G cell lines is reduced to glutathione. Elevated production of GSH is linked to glioblastoma cells but its decrease is connected to a worse prognosis in patients, which could mean a more aggressive tumor [51,52,53]. The quantity of adenosine in cell lysates of U118 cells was higher. At the same time, hypoxanthine, catabolite of adenosine, is released to the U118 culture medium; however, it is taken up from the T98G cell line media. Adenosine is correlated with aggressiveness of glioblastoma cells [54]. All of these could refer to increased metabolic activity in U118 compared to T98G, in addition to even lower GSH levels. High metabolic activity produces reactive oxygen species which could interact with GSH and oxidize it to its oxidized form (GSSH) [47]. Unfortunately, we were not able to estimate the level of GSSH to further support this hypothesis by calculating the ratio of GSH to GSSH.

Moreover, the intracellular content of some of the amino acids was significantly higher, while their uptake from the culture medium was not. Among these are essential amino acids such as tryptophan, histidine, lysine, threonine, or isoleucine as well as non-essential arginine and tyrosine. All of these were elevated in U118 cells. Although arginine and tyrosine could be synthesized in cells, other nonessentials cannot. The source of these amino acids could also be extracellular proteins as in the case of phenylalanine in the culture medium and one cell line could be more aggressive in this process or could be better equipped for the purpose [55]. Additionally, tyrosine can be synthesized from phenylalanine, which was released to the medium, and it can support hypothesis of extracellular protein scavenging.

Based on the obtained data we can postulate that the provided protocol for LC-MS analysis is a useful tool for studies on glioblastoma metabolism and may be used to identify metabolic heterogeneity among cultured glioblastoma cell types. Metabolic heterogeneity of glioblastoma cells in their capability to metabolize glucose is a subject for recent scientific research [3,8,18,19,20].

The limitations in supplementation of glioblastoma cells with glucose in situ, together with the assumption that the transformed metabolism of glioblastoma cells can also engage other substrates, including amino acids [3,9,10,11,12,13], are bases for postulating the question about metabolic heterogeneity of glioblastoma cells regarding substrates other than glucose. Indeed, our results confirm that glioblastoma cells remove some of amino acids from their milieu. However, the increase in the level of phenylalanine, which is an essential amino acid, points to a possibility that glioblastoma cells can also rely on extracellular proteins as a source of amino acids [56].

Several lipidomic studies confirm the heterogeneity of metabolism of lipids and non-polar molecules among glioblastomas [57,58,59]. The limitation of our study is that the HILIC-MS we applied is not suitable for appropriate separation of non-polar molecules and therefore to estimate compound specific retention time.

Glioblastoma is a highly malignant brain tumor that is characterized by its invasive growth and resistance to current therapies. Metabolomics, the study of the small molecule metabolites in biological systems, has emerged as a powerful tool for understanding the metabolic alterations that occur in glioblastoma cells and for identifying potential targets for therapy. Metabolomics studies of glioblastoma have also identified potential metabolic biomarkers that can be used for diagnosis or monitoring of the disease. For example, studies have identified 2-hydroxyglutarate as an oncometabolite, whose level is elevated not only in patients with 2-hydroxyglutaric aciduria but also in the biological fluids of patients with glioblastoma [60,61,62,63,64,65].

Overall, metabolomics studies based on LC-MS have emerged as a valuable tool for understanding the metabolic alterations that occur in glioblastoma cells and for identifying potential targets for therapy. Further research in this area is needed to fully exploit the potential of metabolomics for the development of new therapies for glioblastoma.

## 5. Conclusions

In conclusion, the application of HILIC-MS to investigate the metabolic heterogeneity of cultured glioblastoma cells has provided valuable insights into the transformed metabolism of polar and ionic compounds. Specifically, the analysis of amino acids using HILIC-MS has appeared to be particularly informative, as it has revealed significant alterations in the levels of various amino acids, which might play critical roles in tumor growth and proliferation. So, in addition, to common dysregulated metabolic pathways among different types of glioblastoma cells, which can be exploited as potential therapeutic targets, the use of HILIC-MS for studying the metabolic heterogeneity of glioblastoma cells is a promising approach for advancing our understanding of this complex disease and expansion of the possible spectrum for the therapeutic approaches.

## Figures and Tables

**Table 1:** The list of compounds detected by LC-MS. The common and IUPAC names for detected compounds with a value of *m*/*z* and retention time (t_R_) which were used for identification of the compound chromatogram obtained after separation on Sequant^®^ ZIC^®^-cHILIC column.

Common Name	IUPAC Name	*m*/*z*	t_R_ (min)
Acetoacetate	3-Oxobutanoic acid	101.0258	5.5
Acetylcarnitine	3-Acetyloxy-4-trimethylammonio-butanoate	204.1230	13.3
Acetyl-CoA	O1-{(3R)-4-[(3-{[2-(Acetylsulfanyl)ethyl]amino}-3-oxopropyl)amino]-3-hydroxy-2,2-dimethyl-4-oxobutyl} O3-{[(2R,3S,4R,5R)-5-(6-amino-9H-purin-9-yl)-4-hydroxy-3-(phosphonooxy)oxolan-2-yl]methyl} dihydrogen diphosphate	404.0488	12.3
N-Acetylcysteine	2-Acetamido-3-sulfanylpropanoic aci	164.0914	6.3
Adenosine	2-(6-Amino-9*H*-purin-9-yl)-5-(hydroxymethyl)oxolane-3,4-diol	268.1022	3.7
ADP	[(2R,3S,4R,5R)-5-(6-Aminopurin-9-yl)-3,4-dihydroxyoxolan-2-yl]methyl phosphono hydrogen phosphate	426.0137	11.2
AICA Riboside-5-phosphate	1-(5-Amino-4-carbamoyl-1H-imidazol-1-yl)-1,4-anhydro-D-ribitol 5-(dihydrogen phosphate)	339.0685	10.8
Alanine	2-Aminopropanoic acid	90.055	7.3
Alanyl-Glutamine	5-Amino-2-[[(2S)-2-aminopropanoyl]amino]-5-oxopentanoic acid	218.1508	5.7
γ-Aminobutyric acid	4-Aminobutanoic acid	104.0706	4.6
Arachidonic acid	Icosa-5,8,11,14-tetraenoic acid	303.2324	1.7
Arachidonic acid methyl ester	Methyl-icosa-5,8,11,14-tetraenoic acid	319.2225	1.7
Arginine	2-Amino-5-(diaminomethylideneamino)pentanoic acid	175.119	15.9
Ascorbic acid	3,4-Dihydroxy-5-((S)-1,2-dihydroxyethyl)furan-2(5H)-one	175.0238	12.7
Asparagine	2,4-Diamino-4-oxobutanoic acid	133.0596	10.4
Aspartic acid	2-Aminobutanedioic acid	134.0476	10.9
ATP	[[(2R,3S,4R,5R)-5-(6-Aminopurin-9-yl)-3,4-dihydroxyoxolan-2-yl]methoxy-hydroxyphosphoryl] phosphono hydrogen phosphate	(−) 505.9805	14.0
ATP Betaine	[[(2R,3S,4R,5R)-5-(6-Aminopurin-9-yl)-3,4-dihydroxyoxolan-2-yl]methoxy-hydroxyphosphoryl] phosphono hydrogen phosphate 2-(Trimethylazaniumyl)acetate	507.9996	14.0
		118.0863	1.6
Biopterin	2-Amino-6-(1,2-dihydroxypropyl)-1H-pteridin-4-one	(−) 236.0804	7.2
Biopterin Biotin	2-Amino-6-(1,2-dihydroxypropyl)-1H-pteridin-4-one 2-Oxohexahydro-1H-thieno [3,4-d]imidazol-4-yl]pentanoic acid	238.1131	7.2
		245.0954	8.8
Butyrylcarnitine	3-Butanoyloxy-4-(trimethylazaniumyl)butanoate	232.1543	10.8
Caffeine	1,3,7-Trimethyl-3,7-dihydro-1H-purine-2,6-dion	195.0937	1.8
cAMP	(4aR,6R,7R,7aS)-6-(6-Aminopurin-9-yl)-2-hydroxy-2-oxo-4a,6,7,7a-tetrahydro-4H-furo [3,2-d][1,3,2]dioxaphosphinin-7-ol	(−) 328.0414	9.7
cAMP Carnitine	(4aR,6R,7R,7aS)-6-(6-Aminopurin-9-yl)-2-hydroxy-2-oxo-4a,6,7,7a-tetrahydro-4H-furo [3,2-d][1,3,2]dioxaphosphinin-7-ol 3-Hydroxy-4-(trimethylazaniumyl)butanoate	330.0603	9.7
		227.1125	12.1
Carnitine Carnosine	3-Hydroxy-4-(trimethylazaniumyl)butanoate 2-(3-Aminopropanamido)-3-(3H-imidazol-4-yl)propanoic acid	162.1125	15.6
		227.1125	12.1
Cervonic acid	Docosa-4,7,10,13,16,19-hexaenoic acid	327.2330	2.2
Citric acid	2-Hydroxypropane-1,2,3-tricarboxylic acid	191.0197	21.6
Citrulline	2-Amino-5-(carbamoylamino)pentanoic acid[	176.1029	10.3
Coenzyme A	[[(2R,3S,4R,5R)-5-(6-Aminopurin-9-yl)-4-hydroxy-3-phosphonooxyoxolan-2-yl]methoxy-hydroxyphosphoryl] [(3R)-3-hydroxy-2,2-dimethyl-4-oxo-4-[[3-oxo-3-(2-sulfanylethylamino)propyl]amino]butyl] hydrogen phosphate	357.0813	5.3
Creatine	2-[Carbamimidoyl(methyl)amino]acetic acid	132.0768	2.4
Creatinine	2-Amino-1-methyl-5H-imidazol-4-one	114.0662	8.5
Cystathionine	S-((R)-2-Amino-2-carboxyethyl)-L-homocysteine	223.0742	13.1
Cytosine	4-Aminopyrimidin-2(1H)-one	112.0497	6.4
Decanoylcarnitine	3-Decanoyloxy-4-(trimethylazaniumyl)butanoate	316.2482	7.6
7,8-Dihydrobiopterin	2-Amino-6-(1,2-dihydroxypropyl)-7,8-dihydro-1H-pteridin-4-one	240.1149	7.5
Dimethylarginine	(2S)-5-(Diaminomethylideneamino)-2-(dimethylamino)pentanoic acid	203.1491	10.3
Dimethyllysine	2-Amino-6-(dimethylamino)hexanoic acid	175.1428	17.7
Dodecanoylcarnitine	3-Decanoyloxy-4-(trimethylazaniumyl)butanoate	344.2795	7.4
Dopamine	4-(2-Aminoethyl)benzene-1,2-diol	154.0863	2.3
EDTA	2-[2-[Bis(carboxymethyl)amino]ethyl-(carboxymethyl)amino]acetic acid	(−) 291.0769	12.1
EDTA EGTA	2-[2-[Bis(carboxymethyl)amino]ethyl-(carboxymethyl)amino]acetic acid 2-[2-[2-[2-[Bis(carboxymethyl)amino]ethoxy]ethoxy]ethyl-(carboxymethyl)amino]acetic acid	293.0982	12.1
		381.1511	10.0
Folic acid	2-[[4-[(2-Amino-4-oxo-1H-pteridin-6-yl)methylamino]benzoyl]amino]pentanedioic acid	440.1324	8.7
Fructose-6-phosphate	6-O-Phosphono-α-D-fructofuranose	259.0217	11.7
Fumaric acid	(E)-But-2-enedioic acid	115.0037	17.6
Gabapentin	2-[1-(Aminomethyl)cyclohexyl]acetic acid	172.1332	1.7
Glucose	(3R,4S,5S,6R)-6-(Hydroxymethyl)oxane-2,3,4,5-tetrol	179.0561	7.8
Glucose-6-phosphate	Glucopyranose 6-phosphate	259.0224	19.4
L-Glutamic acid	(2S)-2-Aminopentanedioic acid	148.0604	10.2
L-Glutamine	(2S)-2,5-Diamino-5-oxopentanoic acid	147.0764	9.8
Glutaric acid	Pentanedioic acid	131.0346	12.1
Glutathione	(2S)-2-Amino-5-[[(2R)-1-(carboxymethylamino)-1-oxo-3-sulfanylpropan-2-yl]amino]-5-oxopentanoic acid	308.0906	11.4
Glycerol 1-phosphate	2,3-Dihydroxyprop yl dihydrogen phosphate	171.008	10.9
Guanine	2-Amino-1,9-dihydro-6H-purin-6-one	152.0563	7.1
GMP	[(2R,3S,4R,5R)-5-(2-Amino-6-oxo-1H-purin-9-yl)-3,4-dihydroxyoxolan-2-yl]methyl dihydrogen phosphate	362.0501	11.2
Hexanoylcarnitine	3-Hexanoyloxy-4-(trimethylazaniumyl)butanoate	260.1856	8.9
Histamine	2-(1H-Imidazol-4-yl)ethanamine	112.0869	11.4
Histidine	2-Amino-3-(1H-imidazol-5-yl)propanoic acid	156.0768	14.1
Homocarnosine	2-(4-Aminobutanoylamino)-3-(1H-imidazol-5-yl)propanoic acid	241.1278	12.2
Homocysteine	2-Amino-4-sulfanylbutanoic acid	136.0427	9.8
α-Hydroxyglutaric acid	2-Hydroxypentanedioic acid	147.0293	12.0
3-Hydroxybutyric acid	3- Hydroxypentanedioic acid	103.0401	2.4
D-2-Hydroxyglutaric acid	(2R)-2-Hydroxypentanedioic acid	147.0314	11.4
L-2-Hydroxyglutaric acid	(2S)-2-Hydroxypentanedioic acid	147.0314	11.4
3-Hydroxyisobutyric acid	3-Hydroxy-2-methylpropanoic acid	103.0401	2.4
Hydroxyproline	4-Hydroxypyrrolidine-2-carboxylic acid	132.0998	10.0
Hypoxanthine	1,9-Dihydro-6H-purin-6-one	135.0375	5.7
Inosine	3,4-Dihydroxy-5-(hydroxymethyl)oxolan-2-yl]-6,9-dihydro-3H-purin-6-one	269.0858	6.9
Inosine 5′-monophosphate	[3,4-bis(trimethylsilyloxy)-5-(6-trimethylsilyloxypurin-9-yl)oxolan-2-yl]methyl bis(trimethylsilyl) phosphate	(−) 347.041	10.7
Inosine 5′-monophosphate Inositol	[3,4-bis(trimethylsilyloxy)-5-(6-trimethylsilyloxypurin-9-yl)oxolan-2-yl]methyl bis(trimethylsilyl) phosphate Cyclohexane-1,2,3,4,5,6-hexol	349.1287	10.7
		179.0521	8.9
Isocitrate	1-Hydroxypropane-1,2,3-tricarboxylic acid	191.0197	21.6
Isoleucine	2-Amino-3-methylpentanoic acid	132.1019	7.3
Isovaleryl-CoA	S-{(9R)-1-[(2R,3S,4R,5R)-5-(6-Amino-9H-purin-9-yl)-4-hydroxy-3-(phosphonooxy)tetrahydro-2-furanyl]-3,5,9-trihydroxy-8,8-dimethyl-3,5-dioxido-10,14-dioxo-2,4,6-trioxa-11,15-diaza-3λ5,5λ5- diphosphaheptadecan-17-yl} 2-methylpropanethioate	850.1486	11.1
Itaconic acid	Methylidenebutanedioic acid	129.0219	10.2
α-Ketoisocaproic acid	4-Methyl-2-oxopentanoic acid	(−) 129.0582	2.9
α-Ketoisovaleric acid	3-Methyl-2-oxobutanoic acid	(−) 115.0428	3.3
α-Keto-β-methylvaleric acid	2-formylpentanoic acid	(−) 129.0582	2.9
Kynurenine	2-Amino-4-(2-aminophenyl)-4-oxo-butanoic acid	209.0921	11.2
Lactic acid	2-Hydroxypropanoic acid	89.0244	2.0
Leucine	2-Amino-4-methylpentanoic acid	132.1019	7.5
γ-Linolenic acid	Octadeca-6,9,12-trienoic acid	277.2173	2.2
Linoleic acid	Octadeca-9,12-dienoate	279.233	2.3
Lysine	2,6-Diaminohexanoic acid	147.1128	15.8
Malic acid	2-Hydroxybutanedioic acid	133.0142	17.5
Methionine	2-Amino-4-(methylthio)butanoic acid	150.0583	8.8
3-Methyladenine	3-Methyl-7H-purin-6-imine	157.0774	2.4
Methylcrotonyl-CoA	3′-O-Phosphonoadenosine 5′-[(3R)-3-hydroxy-2-methyl-4-{[3-({2-[(3-methylbut-2-enoyl)sulfanyl]ethyl}amino)-3-oxopropyl]amino}-4-oxobutyl dihydrogen diphosphate]	850.1654	11.3
Methylcrotonyl-CoA 5-Methylcytosine	3′-O-Phosphonoadenosine 5′-[(3R)-3-hydroxy-2-methyl-4-{[3-({2-[(3-methylbut-2-enoyl)sulfanyl]ethyl}amino)-3-oxopropyl]amino}-4-oxobutyl dihydrogen diphosphate] 4-Amino-5-methylpyrimidin-2(1H)-one	(−) 848.1336	11.3
		126.0617	4.2
6-*O*-Methylguanine	6-Methoxy-9H-purin-2-amine	166.0703	3.6
7-Methylguanine	2-Amino-7-methyl-1,7-dihydro-6H-purin-6-one	166.071	5.5
5-Methylthioadenosine	5′-S-Methyl-5′-thioadenosine	298.0958	1.9
Trimethyllysine	(5-Amino-5-carboxypentyl)-trimethylazanium	189.1598	8.5
NAD^+^	5-(6-Aminopurin-9-yl)-3,4-dihydroxyoxolan-2-yl]methoxy-hydroxyphosphoryl] [(2R,3S,4R,5R)-5-(3-carbamoylpyridin-1-ium-1-yl)-3,4-dihydroxyoxolan-2-yl]methyl hydrogen phosphate	665.1215	16.8
NADH	5-(6-Aminopurin-9-yl)-3,4-dihydroxyoxolan-2-yl]methoxy-oxidophosphoryl] [(2R,3S,4R,5R)-5-(3-carbamoylpyridin-1-ium-1-yl)-3,4-dihydroxyoxolan-2-yl]methyl phosphate	666.1337	16.4
Neopterin	2-Amino-6-(1,2,3-trihydroxypropyl)pteridin-4(1H)-one	252.0678	8.9
Neopterin	2-Amino-6-(1,2,3-trihydroxypropyl)pteridin-4(1H)-one	254.1203	8.9
Niacinamide	Pyridine-3-carboxamide	123.057	1.8
N-Methyl-D-aspartic acid	2-(Methylamino)butanedioic acid	(−) 146.0484	7.1
N-Methyl-D-aspartic acid N-Methyllysine	2-(Methylamino)butanedioic acid [(5S)-5-Carboxy-5-(methylamino)pentyl]azanium	148.0605	7.1
		161.1273	16.2
N-Methyl phenylalanine	2-(Methylamino)-3-phenylpropanoic acid	180.1015	7.2
Octanoylcarnitine	3-Octanoyloxy-4-(trimethylazaniumyl)butanoate	288.2169	8.0
Ornithine	2,5-Diaminopentanoic acid	133.0972	15.5
2-Oxoglutaric acid	2-Oxopentanedioic acid	145.0142	16.0
Palmitic acid	Hexadecanoic acid	255.2330	2.3
Palmitoylcarnitine	3-hexadecanoyloxy-4-(trimethylazaniumyl)butanoate	400.3421	7.0
Pantothenic acid	2,4-Dihydroxy-3,3-dimethylbutanamido]propanoic acid	(−) 218.0983	7.4
Pantothenic acid Phenylalanine	2,4-Dihydroxy-3,3-dimethylbutanamido]propanoic acid 2-Amino-3-phenylpropanoic acid	220.1334	7.4
		166.9863	7.2
Phenylbutyric acid	4-Phenylbutanoic acid	163.076	2.2
Phosphocreatine	N-Methyl-N-(phosphonocarbamimidoyl)glycine	210.0258	12.2
Phosphoenolpyruvic acid	2-(Phosphonooxy)prop-2-enoic acid	169.1011	1.4
Proline	Pyrrolidine-2-carboxylic acid	116.0706	8.5
Pyridoxine	4,5-Bis(hydroxymethyl)-2-methylpyridin-3-ol	170.0941	2.7
Pyroglutamic acid	5-Oxopyrrolidine-2-carboxylic acid	130.0449	10.2
Retinol	3,7-Dimethyl-9-(2,6,6-trimethylcyclohex-1-en-1-yl)nona-2,4,6,8-tetraen-1-ol	285.2224	3.9
Riboflavin	7,8-Dimethyl-10-[(2S,3S,4R)-2,3,4,5-tetrahydroxypentyl]benzo[g]pteridine-2,4-dione	377.1488	4.0
Ribose	5-(Hydroxymethyl)oxolane-2,3,4-triol	149.047	9.9
Serotonin	3-(2-Aminoethyl)-1H-indol-5-ol	177.1002	3.2
Stearic acid	Octadecanoic acid	283.2643	2.3
Succinyl-CoA	4-{[1,3-Dihydroxy-1,3-dioxo-3-(3′-O-phosphonoadenosin-5′-O-yl)-1λ5,3λ5-diphosphoxan-1-yl]oxy}-3,3-dimethylbutanamido]propanamido}ethyl)sulfanyl]-4-oxobutanoic acid	117.0198	11.8
Taurine	2-Aminoethanesulfonic acid	126.0231	10.0
Tetradecanoylcarnitine	3-Tetradecanoyloxy-4-(trimethylazaniumyl)butanoate	372.3108	7.2
Tetrahydrobiopterin	2-Amino-6-[(1R,2S)-1,2-dihydroxypropyl]-5,6,7,8-tetrahydropteridin-4(1H)-one	242.1244	7.9
Thiamine	2-[3-[(4-Amino-2-methylpyrimidin-5-yl)methyl]-4-methyl-1,3-thiazol-3-ium-5-yl]ethanol	265.1181	12.8
Threonine	2-Amino-3-hydroxybutanoic acid	120.0655	7.7
Tocopherol	2,5,7,8-Tetramethyl-2-[(4R,8R)-4,8,12-trimethyltridecyl]-3,4-dihydro-2H-1-benzopyran-6-ol	429.3738	2.2
Tryptophan	2-Amino-3-(1H-indol-3-yl)propanoic acid	205.0972	7.8
Tyrosine	2-Amino-3-(4-hydroxyphenyl)propanoic acid	182.0812	20.1
Valine	2-Amino-3-methylbutanoic acid	118.0863	7.7
Vitamin D	9,10-Secocholesta-5,7,10(19)-trien-3-ol	383.3319	3.0
Vitamin K	3,7,11,15-Tetramethylhexadec-2-enyl]naphthalene-1,4-dione	449.3425	4.3
Xanthine	3,7-Dihydro-1H-purine-2,6-dione	151.0285	5.8

(−)—detection of compound in negative mode.

**Table 2:** Retention time (t_R_) values estimated for leucine and isoleucine on tested columns with estimated peak resolution value.

Column	t_R_(ILE) (min)	t_R_(LEU) (min)	Peak Resolution
Sequant^®^ ZIC^®^-cHILIC	7.3	7.5	1.28
Sequant^®^ ZIC^®^ pHILIC	6.7	7.0	1.08
Raptor Polar X	1.3	1.4	1.25
YMC-Triart-Diol-HILIC	7.4	7.5	0.55

**Table 3:** Concentration ranges, linearity, and precision for selected analytes. The linearity represents the value of R^2^ that was estimated by regression analysis for the fit between relative area and analyte concentration. Coefficient of variation for precision was calculated from five measurements of standard sample and is expressed as relative standard error.

Analyte	Concentration Range (nM)	Linearity	Precision (%)
Adenosine	3.7–375	0.9920	5.4
Arginine	27.7–574	0.9664	19.2
Asparagine	7.5–751	0.9615	13.8
Glutamate	34–680	0.9734	13.5
Glutamine	68.4–685	0.9824	8.6
Glutathione—reduced	3.3–325	0.9820	15.9
Histidine	64.4–644	0.9923	30.5
Hypoxanthine	7.3–735	0.9857	8.4
Isoleucine	76.2–762	0.9690	8.7
Leucine	76.2–762	0.9637	8.2
Methionine	6.7–670	0.9718	29.7
5-Methylcytosine	40–800	0.9922	7.6
5-Methylthioadenosine	3.4–335	0.9978	10.2
Phenylalanine	6.1–605	0.9768	8.2
Threonine	8.4–840	0.9735	15.0
Tryptophan	4.9–490	0.9905	13.3

## Data Availability

The research data are available upon request. The data are not publicly available due to privacy concerns.

## Supplementary Materials

The following supporting information can be downloaded at: https://www.mdpi.com/article/10.3390/metabo14060297/s1, Supplementary material consists of Table S1: List of analytes and their precision and Figure S1: Fold-change of metabolites in DMEM cell culture medium.
