# Supplementary material for: Application of the Hydrophilic Interaction Liquid Chromatography (HILIC-MS) Novel Protocol to Study the Metabolic Heterogeneity of Glioblastoma Cells
Source: Metabolites. 2024 May 23;14(6):297. doi: 10.3390/metabo14060297 (PMC11205371; doi:10.3390/metabo14060297)
Supplement: Supplementary file 1 [file metabolites-14-00297-s001.zip › metabolites-2980804-supplementary.pdf]

Table S1. List of analytes and their precision expressed as relative standard errors of metabolites identified in cell culture medium without cells. Results are for 3 independent samples, each injected and analyzed once.

Analyte	Precision (%)
ARG	32.7
Citrulline	10.7
Creatine	14.2
GLC	17.2
GLN	9.9
HIS	6.4
Hypoxanthine	11.4
ILE	11.2
Inositol	8.5
LEU	14.9
LYS	26.3
MET	13.5
5-MTA	7.4
Niacinamide	5.4
Palmitic acid	6.9
Pantothenic acid	11.4
PHE	4.0
PRO	24.6
Pyridoxine	19.4
Riboflavin	14.8
Stearic acid	6.4
Thiamine	5.4
THR	14.4
TRP	4.3
TYR	18.0
VAL	5.1

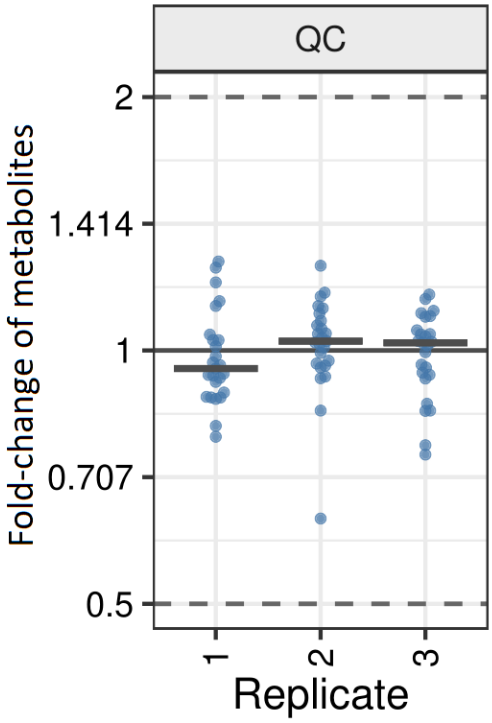

Figure S1. Fold-change of metabolites in DMEM cell culture medium enriched with 10% (v/v) fetal bovine serum. Each point denotes a single metabolite and the variations in its fold-change across the replicates. Figure depicts three individual samples, each injected and analyzed once. QC – Quality control. Graph generated by Metabolite AutoPlotter software.
